# Sacituzumab govitecan expands its therapeutic spectrum among breast cancer subtypes

**DOI:** 10.17305/bb.2022.8874

**Published:** 2023-03-16

**Authors:** Semir Vranic

**Affiliations:** 1 College of Medicine, QU Health, Qatar University, Doha, Qatar

## Introduction

Antibody-drug conjugates (ADCs) are novel, highly potent drugs composed of a small molecule of an anticancer drug (payload) attached to humanized antibody recognizing an epitope on the surface of cancer cells. ADCs are rapidly expanding in the oncology field. By 2022, >180 ADC-based clinical trials have been conducted [1]. Most of these clinical trials are in phases I or II [1]. Several ADCs have been approved and used for the treatment of various malignancies (e.g., brentuximab vedotin (BV) for the treatment of CD30+ lymphomas, trastuzumab emtansine (T-DM1) for advanced/metastatic/or early-stage high-risk HER2-positive breast cancer with residual disease after neoadjuvant treatment) [2]. Among the new ADCs is sacituzumab govitecan (SG)/Trodelvy^®^, a conjugate of anti-Trop-2 antibody and SN-38 payload (an active metabolite of irinotecan). SG has been recently approved by the Food and Drug Administration (FDA) for the treatment of metastatic triple-negative breast (2020) and urothelial carcinomas (2021) [3]. SG activity is based on targeting the trophoblast cell-surface antigen-2 (Trop-2). Trop-2 (or EpCAM/Trop-1) acts as an epithelial-specific cell adhesion molecule, exhibiting a growth-stimulatory effects [4, 5]. It also transduces intracellular calcium signaling, which can occur without extracellular Ca^2+^ [6].

Trop-2 expression was initially described in trophoblasts and fetal tissues, but subsequently, Trop-2 activity has been demonstrated in various solid (e.g., breast, urothelial, ovarian, colorectal, pancreatic, gastric, prostate, cervical, lung, salivary gland, and oral squamous cell carcinomas) and hematologic malignancies (e.g., leukemias and non-Hodgkin lymphomas) [3, 6–9]. In the breast, Trop-2 expression has been observed in different histologic and molecular subtypes, including luminal and triple-negative breast cancers [8, 10–12].

In contrast to its prognostic value [13], the predictive value of Trop-2 expression has not been established yet. Consequently, predictive testing was not done in any of the trials that led to the approval of SG. However, some ongoing clinical trials, like the one exploring the therapeutic effects of SG in patients with endometrial carcinoma (ClinicalTrials.gov Identifier: NCT04251416) set as an inclusion criterion the availability of endometrial carcinoma tissue and “at least 2+ staining for Trop-2” by immunohistochemistry [14]. Similarly, an ongoing clinical trial (ClinicalTrials.gov Identifier: NCT04152499) will explore the therapeutic utility of *SKB264*, a monoclonal anti-Trop-2 antibody, in advanced/refractory cancers with a prior predictive Trop-2 testing by immunohistochemistry [15].

The most recent (third) FDA approval of SG occurred on February 3, 2023. It is related to luminal breast cancers (estrogen receptor-positive [ER+], HER2-negative [HER2−]), the largest (70%) subgroup of breast carcinomas. The approval was based on a phase 3 randomized clinical trial (TROPiCS-02 study) that involved locally advanced/metastatic luminal breast cancers (ER+/HER2−), previously treated with endocrine and at least two additional systemic treatment modalities [16]. In the TROPiCS-02 study, SG exhibited a significant and clinically meaningful overall survival (OS) benefit of 3.2 months compared with single-agent chemotherapy (median OS: 14.4 months vs 11.2 months; hazard ratio [HR] ═ 0.79; 95% CI: 0.65–0.96; *p* ═ 0.02). SG also demonstrated a substantial (34%) reduction risk of disease progression or death (median progression-free survival: 5.5 vs 4.0 months; HR: 0.66; 95% CI: 0.53–0.83; *p* ═ 0.0003). Notably, 21% of patients treated with SG were progression-free at one year compared with 7% of those treated with conventional chemotherapy [16].

## Conclusion

In advanced/metastatic settings, the treatment of luminal breast cancers remains challenging. Endocrine resistance, poor response to conventional chemotherapy and resistance to several targeted treatment modalities, such as PIK3CA and CDK4/6 inhibitors, are commonly seen in daily practice. Therefore, the approval with SG expands the therapeutic options for the largest subpopulation of breast cancer patients.

Ongoing clinical trials targeting Trop-2 protein appear to be more promising since they include predictive testing for Trop-2 expression and a more individualized therapeutic approach.

## References

[1] Yao P, Zhang Y, Zhang S, Wei X, Liu Y, Du C (2022). Knowledge atlas of antibody-drug conjugates on CiteSpace and clinical trial visualization analysis. Front Oncol.

[2] Ferraro E, Drago JZ, Modi S (2021). Implementing antibody-drug conjugates (ADCs) in HER2-positive breast cancer: state of the art and future directions. Breast Cancer Res.

[3] Vranic S, Gatalica Z (2022). Trop-2 protein as a therapeutic target: a focused review on Trop-2-based antibody-drug conjugates and their predictive biomarkers. Bosn J Basic Med Sci.

[4] Munz M, Baeuerle PA, Gires O (2009). The emerging role of EpCAM in cancer and stem cell signaling. Cancer Res.

[5] Zanna P, Trerotola M, Vacca G, Bonasera V, Palombo B, Guerra E (2007). Trop-1 are conserved growth stimulatory molecules that mark early stages of tumor progression. Cancer.

[6] Shvartsur A, Bonavida B (2015). Trop2 and its overexpression in cancers: regulation and clinical/therapeutic implications. Genes Cancer.

[7] Stepan LP, Trueblood ES, Hale K, Babcook J, Borges L, Sutherland CL (2011). Expression of Trop2 cell surface glycoprotein in normal and tumor tissues: potential implications as a cancer therapeutic target. J Histochem Cytochem.

[8] Dum D, Taherpour N, Menz A, Hoflmayer D, Volkel C, Hinsch A (2022). Trophoblast cell surface antigen 2 expression in human tumors: a tissue microarray study on 18,563 tumors. Pathobiology.

[9] Wolber P, Nachtsheim L, Hoffmann F, Klussmann JP, Meyer M, von Eggeling F (2021). Trophoblast cell surface antigen 2 (Trop-2) protein is highly expressed in salivary gland carcinomas and represents a potential therapeutic target. Head Neck Pathol.

[10] Jeon Y, Jo U, Hong J, Gong G, Lee HJ (2022). Trophoblast cell-surface antigen 2 (TROP2) expression in triple-negative breast cancer. BMC Cancer.

[11] Vidula N, Yau C, Rugo H (2022). Trophoblast cell surface antigen 2 gene *(TACSTD2)* expression in primary breast cancer. Breast Cancer Res Treat.

[12] Vranic S, Palazzo J, Sanati S, Florento E, Contreras E, Xiu J (2019). Potential novel therapy targets in neuroendocrine carcinomas of the breast. Clin Breast Cancer.

[13] Zeng P, Chen M-B, Zhou L-N, Tang M, Liu C-Y, Lu P-H (2016). Impact of TROP2 expression on prognosis in solid tumors: a systematic review and meta-analysis. Sci Rep.

[14] A phase II evaluation of sacituzumab govitecan (IMMU-132), an anti-trop-2-SN-38 antibody-drug conjugate, in patients with persistent or recurrent endometrial carcinoma (2023). https://www.clinicaltrials.gov/ct2/show/NCT04251416?cond=Trop-2&draw=2&rank=5.

[15] A phase I-II, first-in-human study of SKB264 in patients with locally advanced unresectable/metastatic solid tumors who are refractory to available standard therapies (2023). https://www.clinicaltrials.gov/ct2/show/NCT04152499?cond=Trop-2&draw=2&rank=3.

[16] Rugo HS, Bardia A, Marmé F, Cortes J, Schmid P, Loirat D (2022). Primary results from TROPiCS-02: a randomized phase 3 study of sacituzumab govitecan (SG) versus treatment of physician’s choice (TPC) in patients (Pts) with hormone receptor–positive/HER2-negative (HR+/HER2-) advanced breast cancer. J Clin Oncol.

